# Author Correction: Norepinephrine potentiates and serotonin depresses visual cortical responses by transforming eligibility traces

**DOI:** 10.1038/s41467-022-31777-4

**Published:** 2022-07-12

**Authors:** Su Z. Hong, Lukas Mesik, Cooper D. Grossman, Jeremiah Y. Cohen, Boram Lee, Daniel Severin, Hey-Kyoung Lee, Johannes W. Hell, Alfredo Kirkwood

**Affiliations:** 1 Mind/Brain Institute, Johns Hopkins University, Baltimore, MD 21218 USA; 2 Department of Neuroscience, Johns Hopkins University, Baltimore, MD 21205 USA; 3 Department of Pharmacology, University of California at Davis, Davis, CA 95616 USA

**Keywords:** Synaptic plasticity, Long-term depression, Long-term potentiation

Correction to: *Nature Communications* 10.1038/s41467-022-30827-1, published online 09 June 2022.

The original version of this Article omitted from the author list the 6th author Daniel Severin who is from the Mind/Brain Institute, Johns Hopkins University, Baltimore, MD, 21218, USA.

Additionally, the Author Contributions was updated to read: “S.H., L.M., D.S., and C.D.G. collected, analyzed, and interpreted the imaging data, B.L. and J.W.H. made the DSPL and DAPA peptides, C.D.G. and J.Y.C. developed optogenetics in the 5HT-ChR2 mice, S.H., H.K.L., and A.K. wrote the manuscript.”

This has been corrected in both the PDF and HTML versions of the Article.

