# Supplementary material for: Norepinephrine potentiates and serotonin depresses visual cortical responses by transforming eligibility traces
Source: Nat Commun. 2022 Jun 9;13:3202. doi: 10.1038/s41467-022-30827-1 (PMC9184610; doi:10.1038/s41467-022-30827-1)
Supplement: Supplementary file 1 — Supplementary Information [file 41467_2022_30827_MOESM1_ESM.pdf]

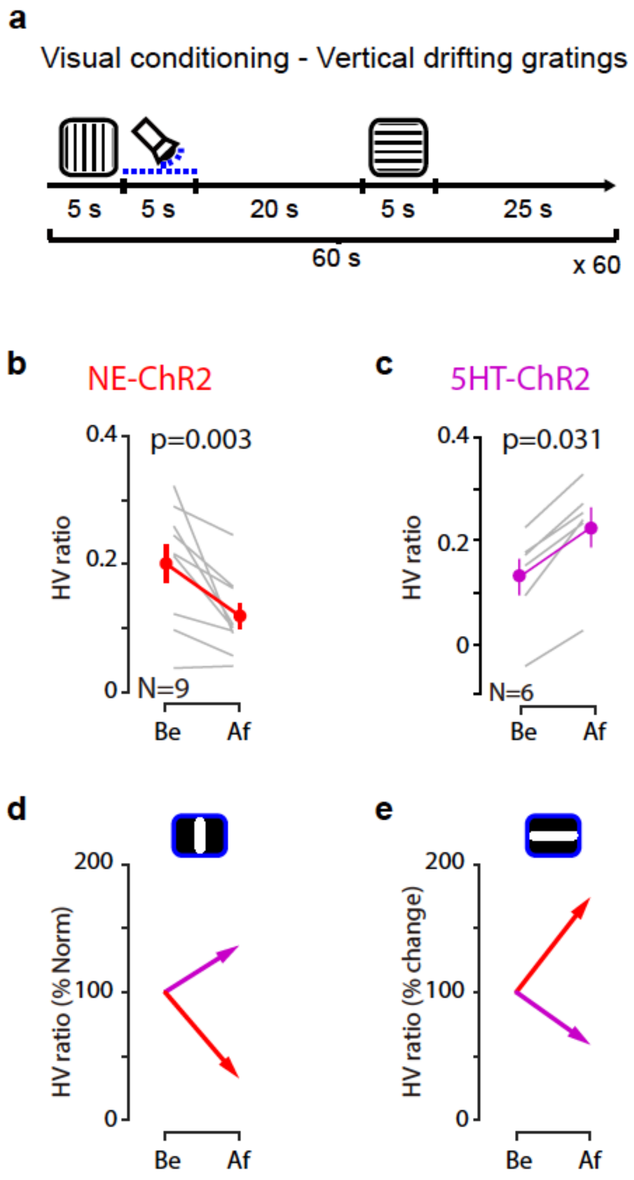

Supplementary Figure 1. Optogenetical reinforcement of V1 responses to vertical drifting gratings.

a Schematics of the conditioning protocol. Imaging of visual cortical responses to vertical and(V) horizontal (H) drifting gratings was performed as in figure1, but with optogenetical stimulation paired to the vertical drifting gratings. **b-c**, Changes in the relative H and V responses to H (HV ratio) recorded before (Be) and after (Af) vertical conditioning in NE-ChR2 mice (**b**) and in 5HT-ChR2 mice (**c**). Thin grey lines: individual mice; thick color lines and symbols: average ± s.e.m. **d-e**, Summary of bidirectional changes in the H/V ratio optogenetical reinforcement of V1 responses to drifting gratings of vertical (**d**) or horizontal (**e**) orientation. In each panel the arrows indicate the average H/V changes (normalized to initial (Be) values) in NE-ChR2 mice (red) and 5HT-ChR2 mice (magenta).

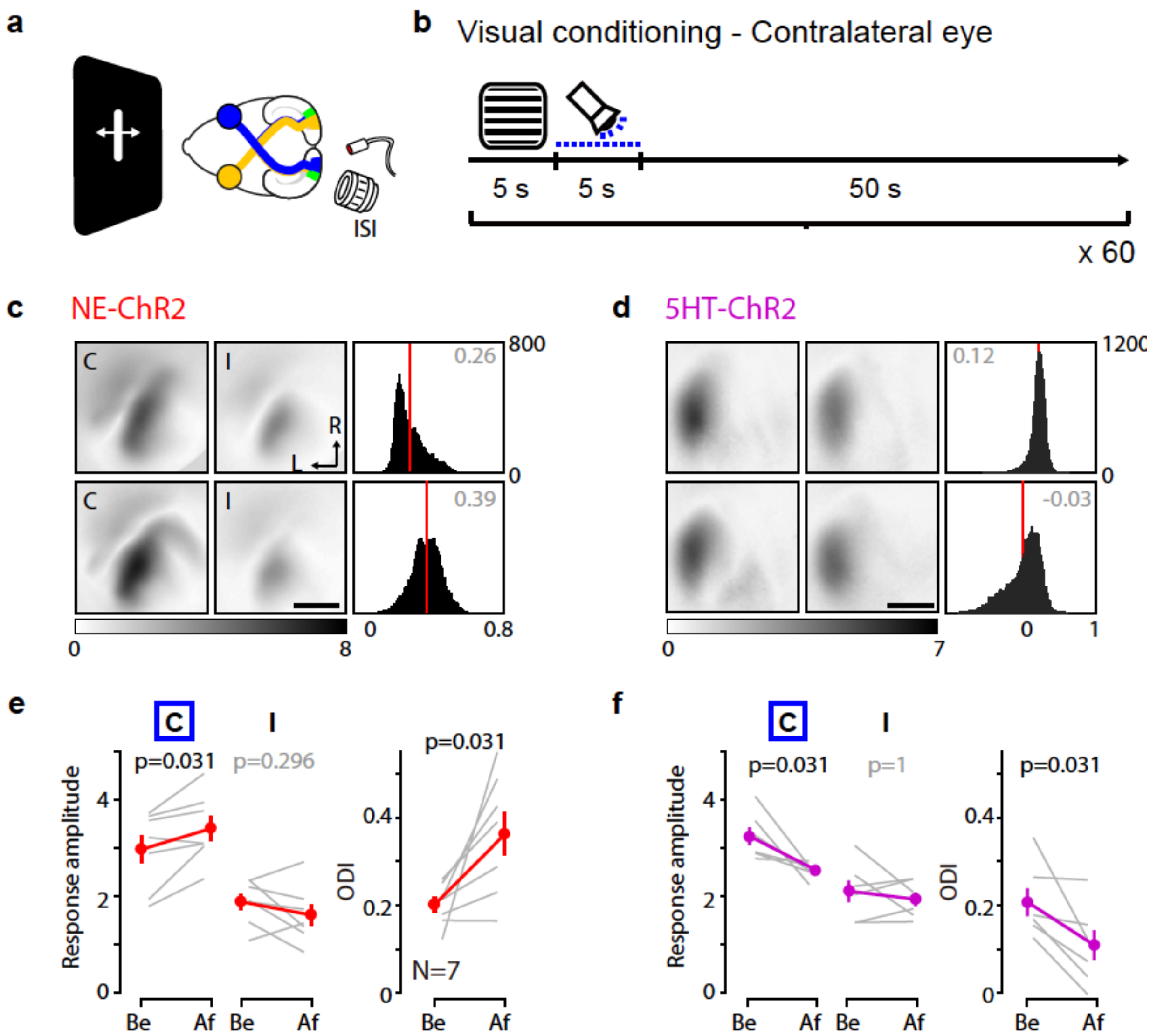

Supplementary Figure 2. Test of input specificity: conditioning one eye does not affect responses of the non-conditioned eye. **a,b** Experimental schematics. **a,** visual responses were imaged in the binocular region (green) of the V1. **b**, Conditioning protocol: the right eye was stimulated 60 times (1 per minute) with horizontal drifting gratings followed by the photoactivation to induce the release of norepinephrine. **c,d**, Example experiments in NE-ChR2 (**c**) and in 5HT-ChR2 mice (**d**). Left: visual cortical responses evoked by the eye contralateral [C] or ipsilateral [I] to the recorded hemisphere. Right: histogram of the ocular dominance index (ODI) illustrated in the number of pixels (x-axis: ODI, y-axis: number of pixels). **e**,**f**, Summary of the changes induced in NE-ChR2 (**e**) and in 5HT-ChR2 mice (**f**). The panels show the changes in response amplitude evoked by the contralateral (C, left) and ipsilateral (I, middle) eye as well as the change of ODI (right) before (Be) and after (Af) the conditioning. Gray scale at bottom of **c,d** represents the fractional change of reflection x10^4Arrows in **c,d**: L, lateral, R, rostral. Scale bar in **c,d**: 1mm.Thin lines in **e,f**: individual experiments; thick lines and symbols: average ± s.e.m.

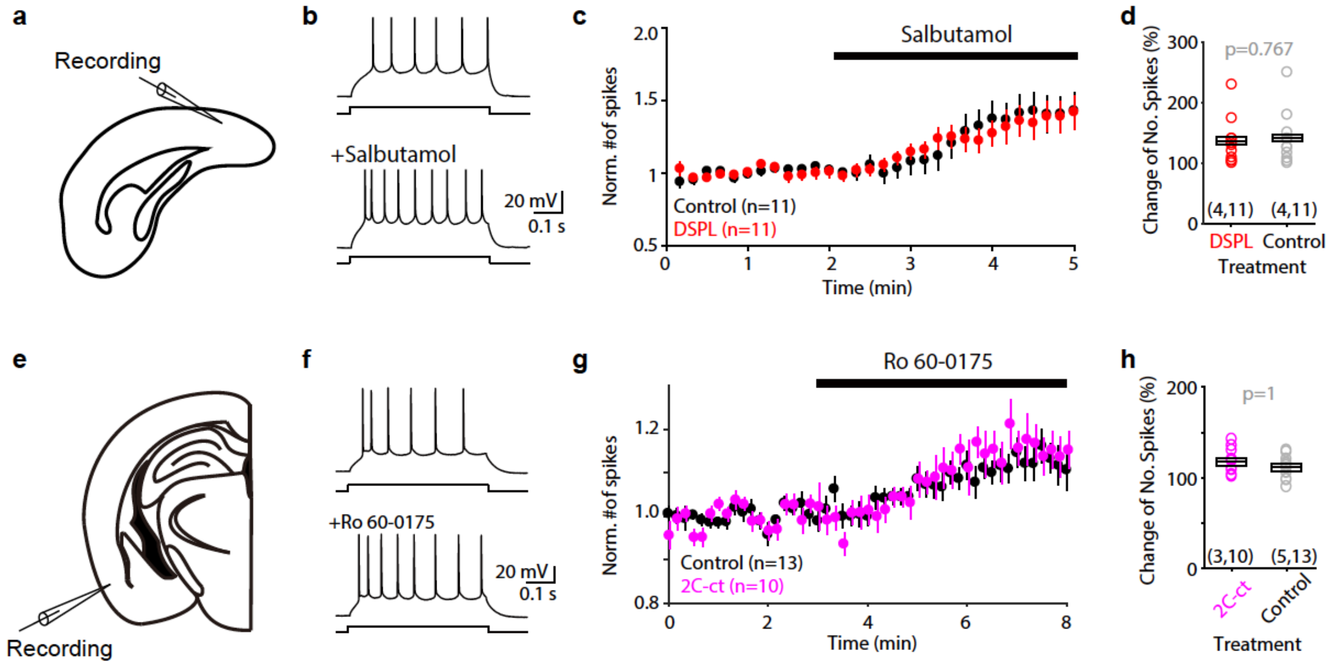

Suplemmentary Figure 3. The DSPL and 2C-ct peptides do not affect the β2AR a nd 5HT2cR effects on intrinsic cellular excitability.

a, Experiment diagram to test the excitability change by the β2AR agonist, salbutamol. The spike response of the layer 2/3 neurons in the V1 were recorded. **b**, Example spike response evoked by current step (bottom of the spike trace, 500 ms) before and after the administration of salbutamol. The current step amplitude was adjusted to evoke 5-6 spikes at the beginning of the recording and then maintained throughout the recording. **c-d**, Summary of the changes of the number of spikes evoked by the current step. The numbers of spikes are normalized by the average number of spikes during the baseline period (0-2 min). Slices were pre-incubated in the ACSF at least 20 min in the absence (black, Control) or presence (red) of DSPL. **e**, Experiment diagram to test the excitability change by the 5HT2cR agonist, Ro 60-0175. V1 neurons did not show excitability change by Ro 60-0175. Therefore, we tested the layer 2/3 neurons in the posterior piriform cortex, where the expression of the 5HT2cR is abundant (Clemett et. al., Neurophrmachology: 39, 123). **f**, Same in (b), but with Ro 60-0175. **g-h**, Same with (c,d), but with 2C-ct. The numbers of spikes are normalized by the average number of spikes during the baseline period (0-3 min). Box plot: average ± s.e.m.

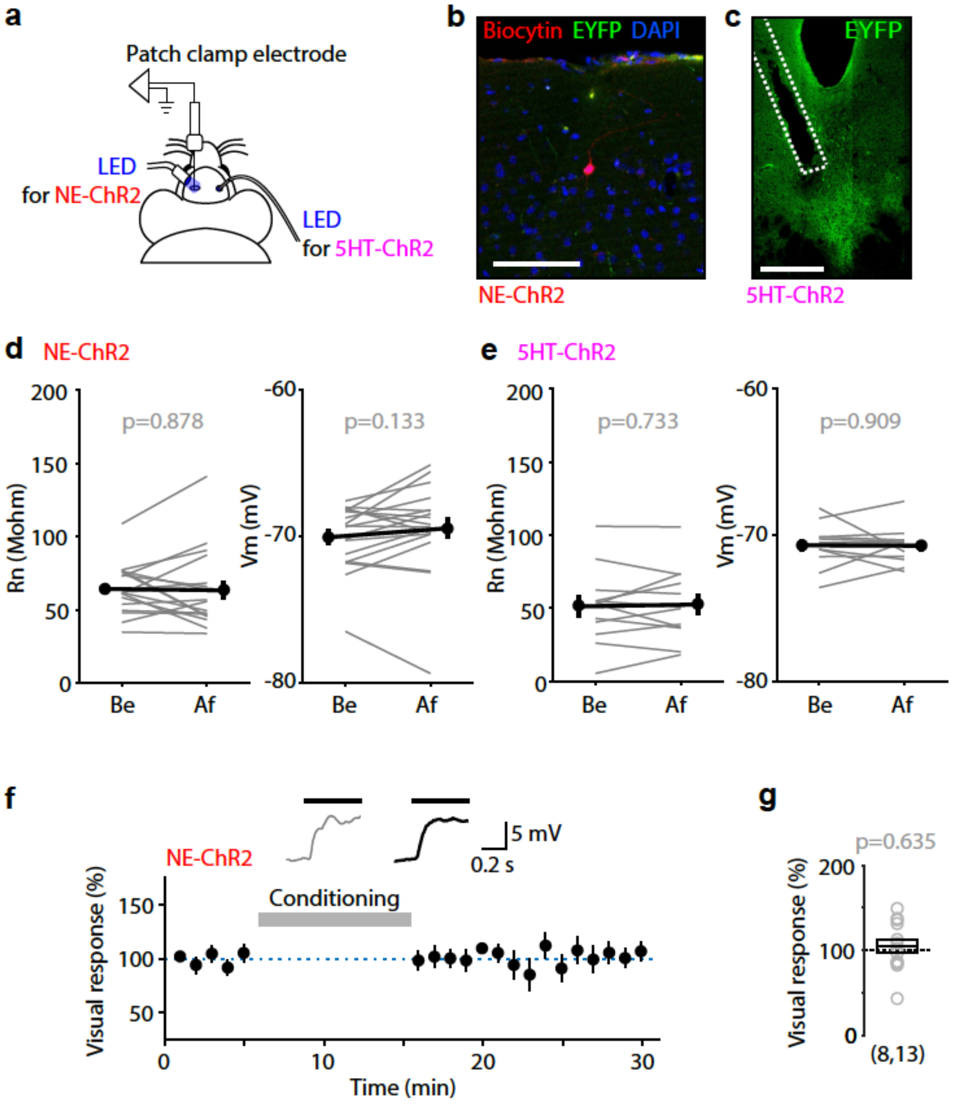

Supplementary Figure 4. In vivo whole cell recording of VEPSPs.
a, Experimental schematics of optogenetic pairing in vivo whole cell recording of VEP SPs. Norepinephrine release was induced by photoactivation of adrenergic neuron ter minal ChR2 of the NE-ChR2 mice at the V1 (left). Serotonin release was induced by p hotoactivation of the virally expressed ChR2 in serotonergic neurons via optic fiber im planted into the dorsal raphe nucleus (DRN) of the 5HT-ChR2 mice (right). **b**, An exa mple fluorescence image from a NE-ChR2 mouse brain slice demonstrating the adren ergic neuronal projections expressing ChR2 (EYFP, green) and the recorded neuron (Biocytin, red). Nuclei are also visualized (DAPI, blue). Scale bar: 100 µm. **c**, An exam ple fluorescence image from a 5HT-ChR2 mouse brain slice demonstrating the ChR2 expression (EYFP, green) in the serotonergic neurons at the DRN. White dotted line in dicates the artifact by the implanted optic fiber. Scale bar: 100 µm. **d-e**, Input resistanc e (Rn) and base membrane potential (Vm) before and after the visual conditioning. **f-g** **,** Normalized change of NC-VEPSPs amplitude of the NE-ChR2 mice by the visual co nditioning without pairing of the postsynaptic spikes (d) and the summary of change (e). Inset traces on top show the VEPSPs of a representative neuron averaged initial (gr ay) or last (black) 5 minutes of the recording. Box plot: average ± s.e.m.

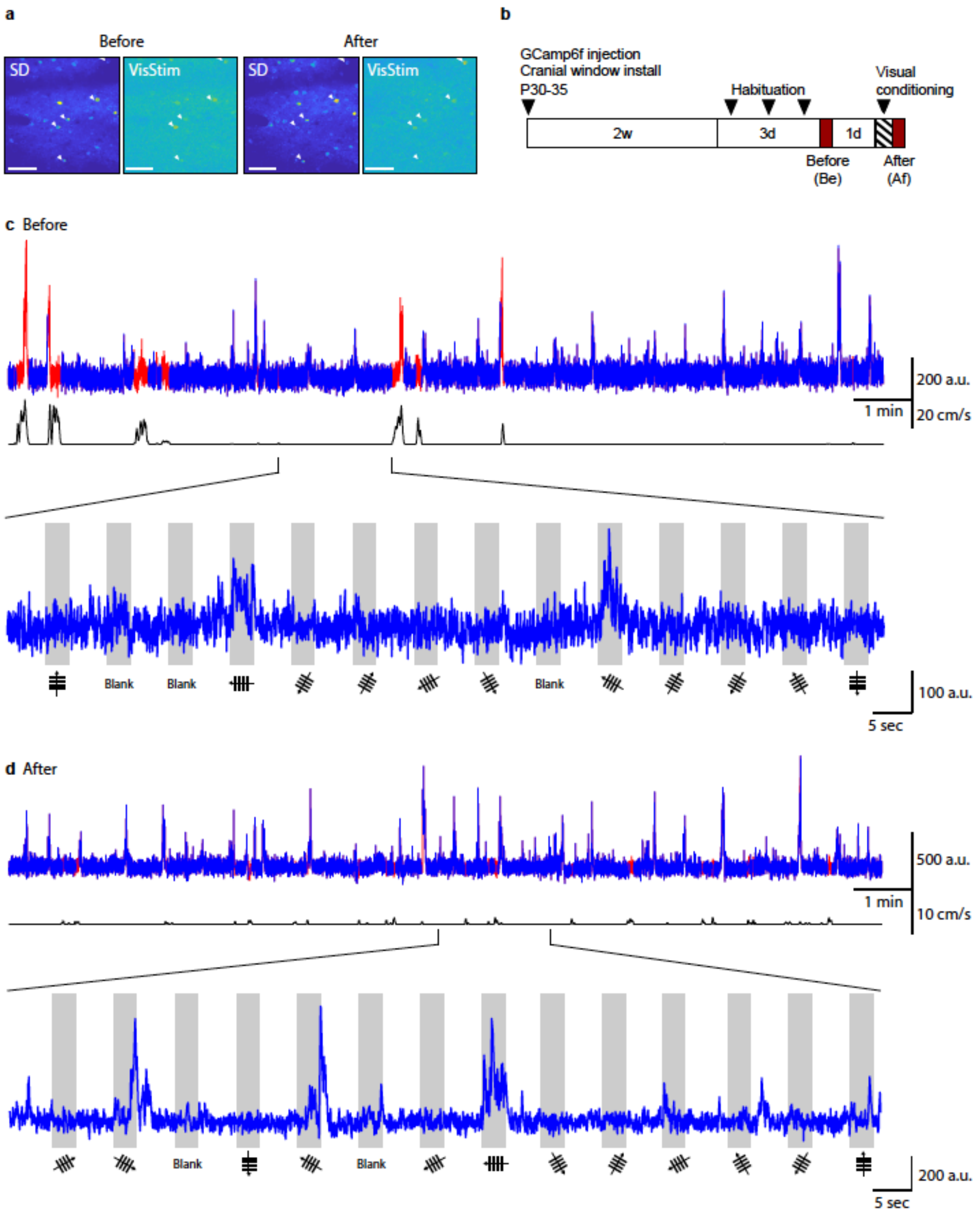

Supplementary Figure 5. Two-photon calcium imaging of superficial layer exci tatory neurons.
a, Example two-photon imaging field of view visualized by fluorescence standard de viation (SD) or fluorescence correlation with the visual stimulus (VisStim) before and after the visual conditioning (see Methods). White arrows indicate the stable visually responsive neurons involved in the analysis. Scale bar: 100 μm. **b**, Experiment timel ine. **c-d**, Top, fluorescence signal of the representative neuron across the recording t ime before (c) and after (d) the visual conditioning. Fluorescence signal during the st ationary (blue) or during the locomotion (red) is illustrated based on the treadmill spe ed (black line). Bottom, a part of the fluorescence signal to 14 consecutive visual sti muli is demonstrated. Gray indicate the time the visual stimuli presented. The directi on and the orientation of the visual stimuli are shown at the bottom.

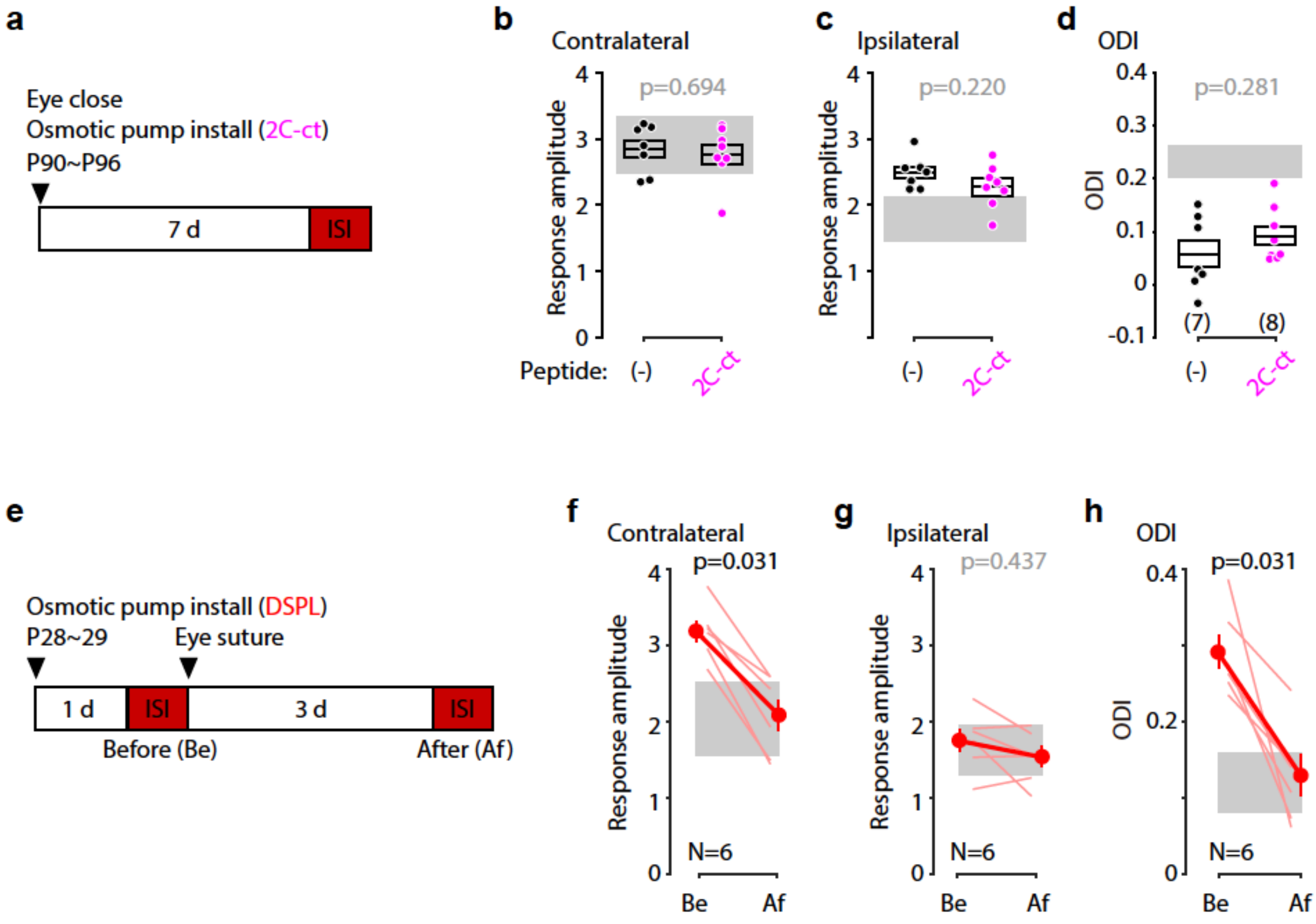

Supplementary Figure 6. Specificity of the disrupting peptides in the interruption o f the LTP or LTD trace transformation.

a-d, Interruption of LTD trace transformation does not impair the potentiation of the open eye by the MD of young adult mice. **a**, Experiment timeline to test the specificity of the 2C -ct. **b-d**, Summary of the changes of the response amplitude evoked by the contralateral (b) and ipsilateral (c) eye as well as the ODI (d) of each experimental group. The data of 7d MD group were previously shown in Fig. 6. Gray region indicates 95% confidential inte rval values of normal reared mice. Box plot: average ± s.e.m. **e-h**, Interruption of LTP trac e transformation does not impair the depression of the closed eye by the MD of juvenile mice. **e**, Experiment timeline to test the specificity of DSPL. **f-h**, Summary of the changes in response amplitude evoked by the contralateral (f) and ipsilateral (g) eye as well as the change of ODI (h) before (Be) and after (Af) the conditioning. Thin line: individual animal; thick line and symbols: average ± s.e.m. Gray areas describe the 95% confidence interva l of the mice deprived for 3 days without the disrupting peptide.
